# pH-responsive polymeric micelles self-assembled from amphiphilic copolymer modified with lipid used as doxorubicin delivery carriers

**DOI:** 10.1098/rsos.171654

**Published:** 2018-03-21

**Authors:** Xin Xin Zhou, Long Jin, Rui Qun Qi, Teng Ma

**Affiliations:** 1 Department of Dermatology, Liaoning University of Traditional Chinese Medicine, Shenyang, People's Republic of China; 2 Second Affiliated Hospital of Liaoning University of Traditional Chinese Medicine, Shenyang, People's Republic of China; 3 Key Laboratory of Ministry of Education for TCM Viscera-State Theory and Applications, Liaoning University of Traditional Chinese Medicine, Shenyang, People's Republic of China; 4 The General Hospital of Shenyang Military, Shenyang, People's Republic of China; 5 Department of Dermatology, No.1 Hospital of China Medical University, Shenyang, People's Republic of China; 6 Key Laboratory of Immunology, Ministry of Public Health of China, Key Laboratory of Immunology, Ministry of Education of China, China Medical University, Shenyang, People's Republic of China; 7 Department of Neurobiology, College of Basic Medicine, China Medical University, Shenyang, People's Republic of China

**Keywords:** pH-sensitive, PAE, micelle, drug delivery, cancer chemotherapy

## Abstract

In the present study, a novel pH-responsive amphiphilic copolymer, 1,2-distearoyl-*sn*-glycero-3-phosphoethanolamine-*N*-[methoxy(polyethylene glycol)] conjugated poly(β-amino esters) (DSPE-*b*-PEG-*b*-PAE-*b*-PEG-*b*-DSPE), was designed and successfully synthesized via Michael-type step polymerization. The chemical structure of the pentablock copolymer was confirmed with proton nuclear magnetic resonance (^1^H-NMR) and Fourier transform infrared (FT-IR) spectroscopy. The copolymer was able to self-assemble into core/shell polymeric micelles in aqueous solution at low concentrations, and its critical micelle concentration (CMC) value was 4.5 mg l^−1^ determined by fluorescence spectrophotometry. The p*K*_b_ value of the copolymer was about 6.5, confirmed by acid–base titration, indicating the pH-sensitivity of the polymeric micelle. The hydrodynamic diameter, distribution and zeta potential of the polymeric micelles at different pH conditions were monitored by dynamic light scattering (DLS). Doxorubicin (DOX) was encapsulated into the core of the micelles with a high drug loading content (15.9%) and entrapment efficacy (60.4%). *In vitro* experiments demonstrated that the release behaviour of DOX from the DOX-loaded polymeric micelles (DOX-PMs) was pH-triggered. When the pH decreased from 7.4 to 5.0, the drug release rate was markedly accelerated. MTT assay showed that the copolymer had negligible cytotoxicity whereas the DOX-PMs displayed high toxicity for tumour cells such as B16F10, HepG2 and HeLa cell lines. The results demonstrated that these pH-sensitive polymeric micelles could be used as potential anti-cancer drug carriers for cancer chemotherapy with controlled release.

## 1. Introduction

To date, various therapeutic strategies for cancer have been emerging and have attracted more and more attention, but chemotherapy is still the most efficient method in clinical practice [1–3]. However, many difficulties and obstacles limit the further clinical application of the traditional chemical drugs such as doxorubicin (DOX), camptothecin and paclitaxel (PTX), such as the severe side-effects, low therapeutic efficacy and serious cytotoxicity [4]. In order to enhance the treatment efficacy and reduce the side-effects, drug delivery systems (DDS) have been developed and used as effective approaches in recent decades, especially polymeric micelles based on functional biomaterials [5–9]. The polymeric micelles self-assembled from amphiphilic polymers not only encapsulate hydrophobic drugs in the core via hydrophobic interaction but also control the release profile of the drugs by responding to stimuli of the tumour microenvironment, such as pH, enzymes and temperature [10–12]. In addition, the polymeric micelles can maintain a stable nano-topological structure and suitable diameter size, which results in extended circulation time in the body and improved accumulation at tumour sites by an enhanced penetration and retention (EPR) effect [13–15]. Consequently, the therapeutic nanoparticles could release encapsulated cargos dependent on the changes of the tumour microenvironment, [16] such as an acidic pH value, higher temperature or specific enzyme, in comparison to normal physiological conditions [16–20].

So far, pH-sensitive polymeric micelles are among the most promising stimuli-responsive carriers for drug delivery and controlled release for cancer chemotherapy [21–24]. It has been reported that the extracellular pH value in most solid tumours is weakly acidic, approximately pH 6.5–7.2, and the intracellular organelles exhibit much lower pH values (pH 4.0–6.5) compared to the pH of 7.4 in normal physiological tissue. Various kinds of pH-responsive polymeric micelles have been developed in recent years [25–28]. For example, poly(*β*-amino ester) (PAE) is a type of polycationic polymer which exhibits a reported p*K*_b_ value of about 6.2 [10,29–31]. When the pH is higher than the p*K*_b_, the PAE is deprotonated and insoluble in water. However, when the pH is lower than the p*K*_b_, the PAE is able to be protonated sequentially and becomes soluble because of the ionization of the amine residues. This physico-chemical property of PAE provides a significant potential in pH-triggered controlled drug release. Zhang *et al.* [4] synthesized a series of amphiphilic pH-responsive copolymers with different PLA/PAE block ratios (mPEG-*b*-(PLA-*co*-PAE)) which can self-assemble into polymeric micelles to use as vehicles for the anti-cancer drug doxorubicin. The DOX-loaded polymeric micelles showed high antiproliferation capacity against HepG2 cells as well as high biocompatibility, indicating the potential application in drug delivery and cancer targeting chemotherapy. Zhao *et al.* [32] developed mixed polymeric micelles self-assembled from stearate-modified hyaluronic acid (SHA), mPEG-*b*-poly(*β*-amino ester) (mPEG-*b*-PAE), and ethylene acetyl-b-poly(*β*-amino ester) (EA-*b*-PAE) which were used for PTX targeted delivery and controlled release. The mixed PTX-loaded polymeric micelles displayed significant enhanced drug accumulation at tumour sites and high cytotoxicity against SKOV-3 cells, demonstrating that these polymeric micelles could be efficient anti-cancer therapeutics in the treatment of ovarian cancer [32]. Furthermore, many therapeutic formulations have been developed and investigated worldwide in preclinical or clinical studies [33,34]. For example, PLGA/goserelin acetate (Zoladex) was first approved by the FDA for treating prostate and breast cancer in 1989, PEGylated liposomal doxorubicin (Doxil) was approved for various types of cancer in 1995, and an albumin/paclitaxel system (Abraxane) was approved for breast cancer in 2005 [35].

**Figure 1.**
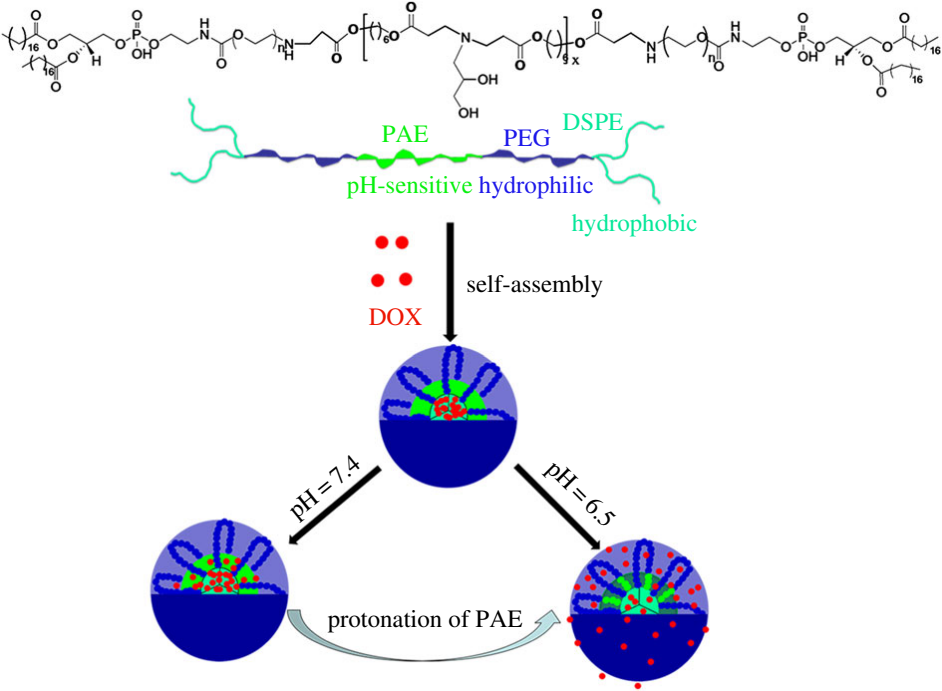
Schematic of self-assembly process and pH-triggered drug release from DSPE-*b*-PEG-*b*-PAE-*b*-PEG-*b*-DSPE polymeric micelles.

In this work, we designed and synthesized a novel amphiphilic pH-sensitive copolymer containing PEGylated lipid and PAE block, and then prepared the polymeric micelles which were used as carriers for anti-cancer drug delivery and controlled release. Firstly, the pH-sensitive PAE monomer capped with diacrylate esters was synthesized via Michael-type step polymerization, and then the amine-ended PEGylated 1,2-distearoyl-*sn*-glycero-3-phosphoethanolamine-*N*-[amino(polyethylene glycol)] (DSPE-PEG-NH_2_) was conjugated on the two terminals, resulting in an amphiphilic copolymer, DSPE-*b*-PEG-*b*-PAE-*b*-PEG-*b*-DSPE, which was able to self-assemble into polymeric micelle in aqueous solution. PEG was hydrophilic and widely used to form the shell, being distributed on the surface of the micelles to reduce reticuloendothelial system (RES) clearance and renal filtration [36–38]. The DSPE containing a long aliphatic chain exhibited high hydrophobicity and formed the micellar core together with PAE during the process of self-assembly, in order to enhance the drug loading capacity and improve the entrapment efficacy [39,40]. Additionally, the DSPE was biocompatible and biodegradable as well being reported to have low cytotoxicity [41,42]. DOX, known to have wide range of applications in cancer chemotherapy, was selected as the small molecular hydrophobic anti-cancer model drug. Figure 1 shows the micellization of the synthesized copolymer and pH-triggered drug release process in the buffer solution. The DOX molecules are efficiently encapsulated in the DSPE and PAE core by hydrophobic interaction. The DOX-loaded polymeric micelles, denoted as DOX-PMs, retained stability and compactness during prolonged blood circulation times under normal physiological conditions. In slightly acidic conditions, the tertiary amine groups of the PAE segment could be ionized, leading to swelling of the polymeric micelles and resulting in accelerated drug release rate from the DOX-PMs. The copolymers should display very little or no cytotoxicity, whereas the DOX-PMs should show high cytotoxic effect against tumour cells. The physico-chemical properties of the copolymer and polymeric micelles, including CMC value, particle size and zeta potential, pH-sensitivity, etc. should be well evaluated by various experimental techniques.

## 2. Material and methods

### 2.1. Materials

1,6-Hexanediol diacrylate (HDD, 99%), (±)-3-amino-1,2-propanediol (AP, 99%), anhydrous dimethyl sulfoxide (DMSO) and dichloromethane (DCM) were obtained from Sigma-Aldrich. 1,2-Distearoyl-*sn*-glycero-3-phosphoethanolamine-*N*-[amino(polyethylene glycol)] (DSPE-PEG-NH_2_, > 99%, *M*_w_ = 2790) was purchased from Avanti Polar Lipids, Inc. (Alabama, USA). Triethylamine (TEA, >99%, Sigma-Aldrich) was further purified and distilled before used. Doxorubicin hydrochloride (DOX-HCl) was purchased from Wuhan Yuan Cheng Gong Chuang Co. and hydrochloric acid was removed before use. Dulbecco's modified Eagle growth medium, fetal bovine serum (FBS), trypsin, penicillin and streptomycin were all purchased from Invitrogen. B16F10, HepG2 and HeLa cell lines were obtained from the Shanghai Institutes for Biological Sciences Cell Resource Center (Shanghai, China) and cultured under the recommended conditions. All other reagents were used as received.

### 2.2. Synthesis of pH-sensitive acrylate-terminated PAE monomer

Acrylate-terminated PAE monomer was synthesized via Michael-type step polymerization as reported elsewhere [10] with a few modifications. A flame-dried 20 ml Schlenk flask with a magnetic stirring bar was firstly evacuated and flushed with nitrogen for three times at room temperature. HDD (1.2 mol) was used as diacrylate and injected into the flask with gently stirring. AP (1.0 mol) was selected as primary amine and slowly added into the flask dropwise. The reaction was carried out for 4 h at 90°C under nitrogen. Subsequently, the mixed solution was cooled down to ambient temperature, and 20 ml of anhydrous DCM was added to dissolve the product. After rotary evaporation, the resulting solution was precipitated in excess cold *n*-hexane three times. The solids were finally dried under vacuum for 48 h to yield the acrylate-terminated PAE, obtained as a solid (greater than 80% yield).

### 2.3. Synthesis of pentablock copolymer DSPE-*b*-PEG-*b*-PAE-*b*-PEG-*b*-DSPE

Amine-ended PEGylated lipid (DSPE-PEG-NH_2_) was used to cap the acrylate-terminated PAE monomer. In brief, DSPE-PEG-NH_2_ (2 mol) and PAE (1 mol) were dissolved into anhydrous DMSO (25 ml), and the mixed solution was transferred to a round bottom flask under nitrogen. The reaction mixture was kept at 45°C with stirring for 48 h. After dialysis and lyophilization, the pentablock copolymer (light yellow powder) was obtained (68% yield).

### 2.4. Characterization of the copolymer

The chemical structures of monomer and copolymer were confirmed by ^1^H-NMR and FT-IR. For ^1^H-NMR measurement, the sample was analysed using an AVANCE III 400 (Bruker, Fällande, Switzerland) at 25°C using deuterated chloroform (CDCl_3_-*d*) as a solvent. For FT-IR measurement, samples were prepared with potassium bromide (KBr) and pressed into thin and round tablets. The measurement was performed using an FT-IR spectrometer (NEXUS 670, Nicolet, USA) at room temperature in the range between 4500 cm^−1^ and 500 cm^−1^, with a resolution of 2 cm^−1^ and 20 scans. The molecular weight (*M*_w_) was determined by gel permeation chromatography (GPC) using an Agilent 1200 series GPC system equipped with an LC quant pump, PL gel 5 mm, 500 Å, 10 000 Å and 100 000 Å column and RI detector. HPLC grade THF was used as a mobile phase with a flow rate of 1.0 ml min^−1^ at 30°C. The column system was calibrated with a set of monodisperse polystyrene standards.

To confirm the pH-sensitivity of the copolymer DSPE-*b*-PEG-*b*-PAE-*b*-PEG-*b*-DSPE, acid–base titration was used to determine the p*K*_b_ value [43]. Briefly, DSPE-*b*-PEG-*b*-PAE-*b*-PEG-*b*-DSPE (10 mg) was dissolved in deionized water (10 ml) in a beaker with stirring, and the pH value of the solution was adjusted to around 3.0 using HCl solution. Subsequently, NaOH solution (0.1 mol ml^−1^) was added to the copolymer solution at increments of 100 µl, and the pH change of the mixed solution was determined by the pH titrator at room temperature.

To confirm the formation of polymeric micelles self-assembled from the pentablock copolymer, the critical micellar concentration (CMC) value of the copolymer DSPE-*b*-PEG-*b*-PAE-*b*-PEG-*b*-DSPE was determined by the fluorescence probe technique using pyrene as a fluorescence probe [4]. The pyrene solution (12 × 10^−5^ M) was prepared first, and 0.1 ml was placed into a vial. The copolymer was dissolved into acetone and deionized water successively at a concentration of 0.1 mg ml^−1^. After evaporation of the acetone with stirring overnight, the pyrene and copolymer solutions were mixed; the copolymer solutions were diluted with deionized water to prepare a range of concentrations (0.0001 mg ml^−1^ to 0.1 mg ml^−1^), and the final concentration of pyrene was 6 × 10^−7^ M in deionized water for every sample. All samples were kept in the dark overnight for equilibration before measurement. The fluorescence excitation spectra of the samples were obtained using a fluorescence spectrophotometer (F-4500, Hitachi, Japan).

### 2.5. Preparation and characterization of DOX-loaded polymeric micelles

The blank and DOX-loaded polymeric micelles, denoted as PMs and DOX-PMs, were prepared by dialysis methods. Briefly, different amounts of DOX-HCl (0, 5, 10, 20 mg) were first dissolved in DMF (40 ml), and excess TEA (approx. 10 µl per 10 mg) was added. The solution was stirred for 1 h in the dark to remove the HCl. The hydrophilic drug DOX-HCl was transformed to hydrophobic drug DOX. Subsequently, copolymer (40 mg) was added to the solution with stirring. The resulting clear mixture was transferred into the pre-swollen cellulose membrane bag (MWCO 3500–4000), and was dialyzed against 1 l deionized water in a beaker for 2 days at room temperature. The external deionized water was replaced every 2 h in the first 12 h and every 6 h in the following 36 h. After filtration through a 0.45 μm filter and lyophilization, the PMs and DOX-PMs were obtained for the study. The drug loading content (LC) and entrapment efficacy (EE) were confirmed by UV–vis spectrophotometry (UV-2450, Shimadzu, Kyoto, Japan) at 481 nm and calculated according to the standard curve of DOX. LC was determined as the weight ratio of drug loaded into the micelles to total drug-loaded micelles. EE was determined as the weight ratio of drug loaded into the micelles to drug in feed.

The zeta potential, hydrodynamic diameter and distribution of the prepared polymeric micelles were measured using dynamic light scattering (DLS; Malvern Zetasizer Nano S, Malvern, UK). In a typical experiment, 1 mg of micelles was dissolved in different buffer solutions and incubated for 2 h. After filtration, the sample was measured in a 1.0 ml quartz cuvette using a diode laser of 800 nm and scattering angle of 90° at 25°C. To evaluate the serum stability, the polymeric micelles were dispersed in the presence of 20% FBS in PBS solution at a final concentration of 1 mg ml^−1^, and the solution was incubated at 37°C for different times to test its stability via diameter changes. The morphology of the micelles was investigated using a transmission electron microscope (TEM, Hitachi H-7650, Tokyo, Japan). Briefly, the polymeric micelle solutions (1 mg ml^−1^) were deposited onto copper grids coated with carbon. The grids were kept at atmospheric pressure and room temperature for 1–2 h for the sticking of samples onto the copper grid, and then the superfluous solution was absorbed by filter paper.

### 2.6. *In vitro* release of DOX from polymeric micelles

The *in vitro* release profiles of DOX from the DOX-PMs were studied using a dialysis bag (MWCO 3500 Da) at 37°C in a dissolution tester (RCZ-8B, TDTF, China) according to Yang *et al.* [43], with a few modifications. Briefly, 4 mg of DOX-PMs was dispersed in 4 ml of respective PBS buffer solutions and placed in a dialysis bag, followed by immersion in 46 ml of PBS solution (pH 7.4, 6.5 and 5.0) in a beaker. The beaker was kept in a water bath with gentle stirring (110 r.p.m.). Samples were withdrawn at desired time intervals and analysed according to Zhang *et al.* [44].

### 2.7. Cytotoxicity test

The *in vitro* cytotoxicity of free drug, PMs and DOX-PMs against B16F10, HepG2 and HeLa cells were evaluated by the standard methylthiazoltetrazolium (MTT) assay. In brief, tumour cells were seeded in a 96-well plate at an initial density of 1 × 10^4^ cells per well. After 1 day, the cells were treated with 200 µl well^−1^ of free DOX, PMs and DOX-PMs in a concentration gradient with fresh medium as control. After incubation for 24 h or 48 h, 20 µl of MTT solution was added to the cultured supernatants, and then the plate was shaken at 150 r.p.m. for 10 min and incubated for a further 4 h. The medium was removed and an equal amount of DMSO was added. Finally, the absorbance at 490 nm was measured using a microplate reader. The cell viability was calculated as the ratio of the difference between sample and blank to the difference between control and blank.

### 2.8. Statistical analysis

The experimental data (mean ± standard deviation (s.d.)) and statistical significance were presented as average values and determined by Student's *t*-test (Excel, 2007), respectively.

## 3. Results and discussion

### 3.1. Synthesis and characterization of DSPE-*b*-PEG-*b*-PAE-*b*-PEG-*b*-DSPE

**Figure 2.**
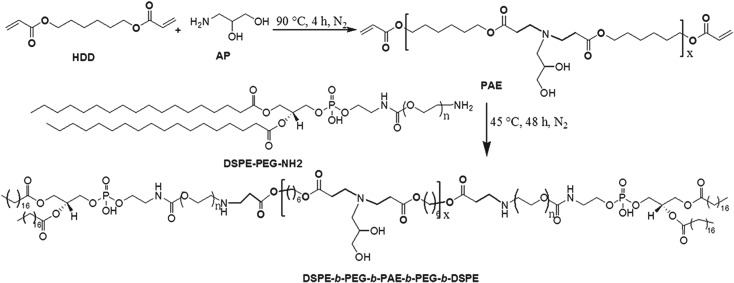
Scheme illustration of the synthesis of pentablock copolymer DSPE-*b*-PEG-*b*-PAE-*b*-PEG-*b*-DSPE.

**Figure 3.**
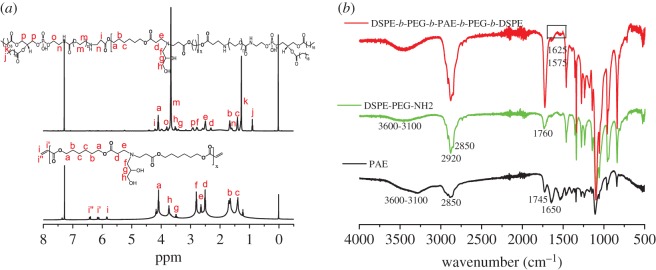
^1^H-NMR (*a*) spectra of diacrylate-terminated polymer PAE and copolymer DSPE-*b*-PEG-*b*-PAE-*b*-PEG-*b*-DSPE in *d*-CDCl_3_, and the according FT-IR (*b*) spectra.

To obtain the amphiphilic pentablock copolymer which could self-assemble into polymeric micelles and respond to the acidity of targeted sites, we first synthesized pH-sensitive poly(*β*-amino esters) (PAE) with diacrylate esters on the two terminals using Michael-type step polymerization. As shown in figure 2, HDD and AP were used as diacrylate esters and diamine at the ratio of 1.2 : 1 (mole/mole), respectively, to synthesize the acrylate-terminated PAE monomer. The double bonds of PAE were then capped with amine-ended PEGylated lipid (DSPE-PEG-NH_2_), resulting in the designed pH-sensitive pentablock copolymer. The chemical structures of the intermediate PAE and copolymer DSPE-*b*-PEG-*b*-PAE-*b*-PEG-*b*-DSPE were confirmed by ^1^H-NMR and FT-IR, as shown in figure 3.

As seen from the ^1^H-NMR spectra of diacrylate-terminated PAE (figure 3*a*, bottom), the signals at 4.07 (a), 1.71 (b) and 1.42 (c) ppm were ascribed to the –O–C*H*2–CH2–CH2–, –O–CH2–C*H*2–CH2– and –O–CH2–CH2–C*H*2– units in PAE, respectively. The three peaks at 2.51–2.83 (d, e, f) ppm were the characteristic resonances from –CO–C*H*2–C*H*2–N–C*H*2– units. The signals at 3.58 (g) and 3.75 (h) were ascribed to the pendant –CH2–C*H*–CH2–OH and –CH2–CH–C*H*2–OH units. The three characteristic peaks at 5.85 (i), 6.14 (i′) and 6.43 (i′′) were ascribed to the three protons of double bonds (C=C) on the two terminals. The results revealed that the diacrylate-terminated PAE polymer was successfully synthesized. After the conjugation of DSPE-PEG-NH_2_ on the terminals of PAE, the three characteristic peaks (i, i′ and i′′) disappeared (figure 3*a*, upper). Additionally, the characteristic peaks of PEG and DSPE were observed at 3.72 (m), 0.89 (j), 1.29 (k) and 3.11 (p) ppm, respectively. In the FT-IR spectra (figure 3*b*), for PAE monomer, the broad and strong peak in the region of 3600–3100 cm^−1^ corresponded to hydroxyl groups (–OH), and the peak of 1650 cm^−1^ corresponded to the C=C stretching vibration band on the terminals. For DSPE-PEG-NH_2_, the peaks of 3600–3100 cm^−1^ were ascribed to the combined peaks of the O–H and N–H stretching vibrations, and the two peaks at 2920 and 2850 cm^−1^ were from the –CH_3_ and –CH_2_– vibrations. The characteristic peak of C = O was observed around 1760 cm^−1^. After conjugation, characteristic peaks of the amide band were found at 1625 and 1575 cm^−1^, and the characteristic peak (1650 cm^−1^) of the C=C bond in PAE was disappeared. All the results demonstrated that the designed amphiphilic pentablock copolymer was synthesized successfully.

### 3.2. pH-sensitivity of copolymer

**Figure 4.**
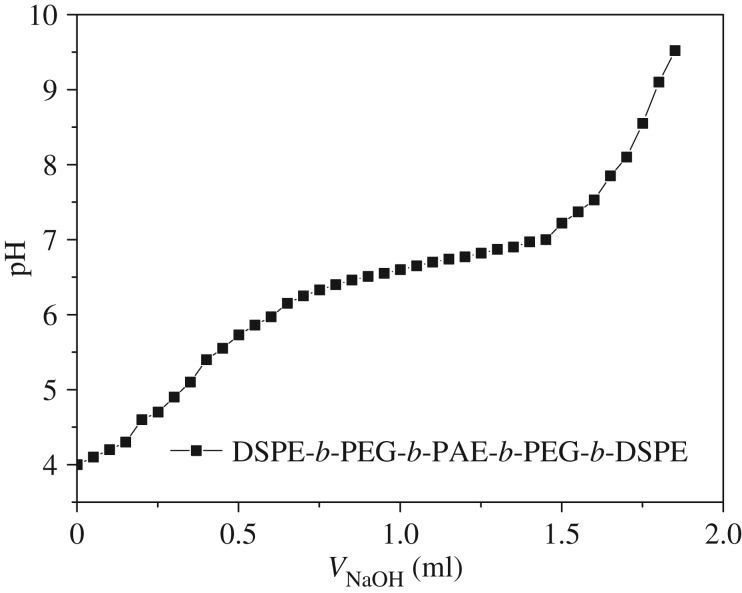
The potentiometric titration of the pentablock copolymer solution.

The pH-sensitivity of the synthesized copolymer was evaluated by acid–base titration, and the corresponding titration curve is shown in figure 4. As expected, the pH value increased sharply with increasing the base content, and then reached a plateau because of the deprotonation/protonation of the tertiary amine residues in the PAE segment. Subsequently, the pH value increased rapidly with the further addition of NaOH solution. In this titration process, the plateau (pH 6.1–6.8) was considered to be the result of the transformation of PAE from a state of deprotonation to protonation. The p*K*_b_ of the polymer was determined as the solution pH at 50% neutralization of the tertiary amine groups, as was reported in the literature [11], and the p*K*_b_ of the synthesized copolymer was confirmed as 6.5. The results indicated that the PAE segment could transform from being hydrophobic to hydrophilic when the pH value was lower than the p*K*_b_, owing to the ionization of tertiary amines.

### 3.3. Micelle formation

**Figure 5.**
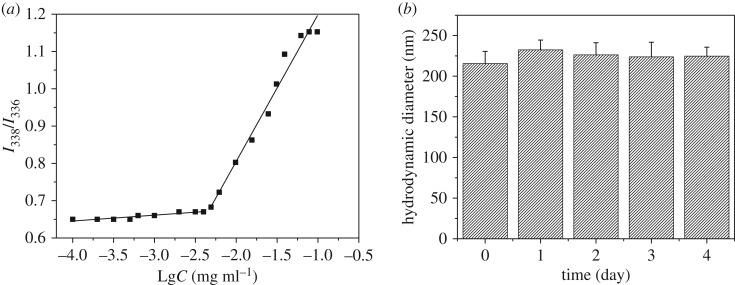
(*a*) Plot of intensity ratios (*I*_338_/*I*_336_) as function of logarithm of the copolymer DSPE-*b*-PEG-*b*-PAE-*b*-PEG-*b*-DSPE concentrations. (*b*) The serum stability of nanoparticles incubated in PBS solution with 20% FBS at pH of 7.4.

To investigate the stability of the polymeric micelles, the CMC value of the copolymer was confirmed by fluorescence spectroscopy using pyrene as the probe. The CMC value not only affected the initial release amount of the entrapped drug (burst release) but also determined the stability of the therapeutic nanoparticles during extended circulation in the body. In the present study, figure 5*a* shows the CMC value of the copolymer. The ratios of *I*_338_ to *I*_336_ were changed with the concentration gradient of copolymer. The intersection of the fitting curves, corresponding to 4.5 mg l^−1^, was the CMC value, indicating that the polymeric micelles could be formed at low polymer concentration. In order to further evaluate the serum stability of the polymeric micelles, the change of particle size was monitored in the presence of 20% FBS in PBS at pH 7.4, as shown in figure 5*b*. After incubation for 4 days, the size change was less than 20%, which indicated that the polymeric micelles were stable in the bloodstream. In summary, the amphiphilic copolymer was able to self-assemble into polymeric micelles even at low polymer concentration, and the polymeric micelles exhibited high stability.

### 3.4. Characterization of blank and DOX-loaded polymeric micelles

**Figure 6.**
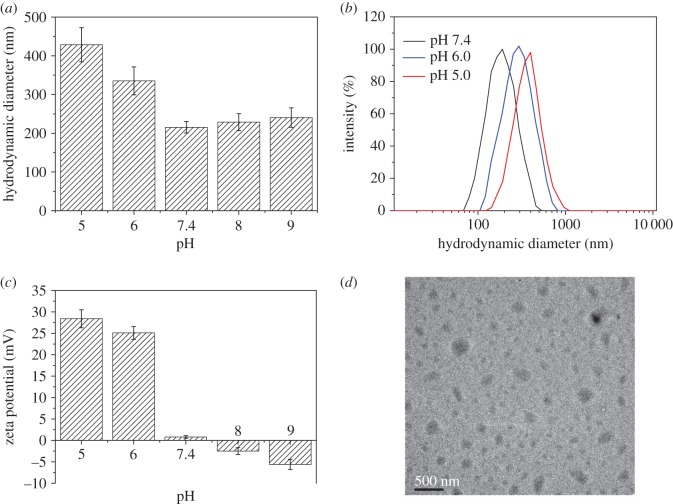
Hydrodynamic diameter (*a*), particle size distribution (*b*) and zeta potential (*c*) of blank polymeric micelles dependent on pH value. TEM image (*d*) of blank polymeric micelles at pH of 7.4.

To investigate the pH-sensitivity of the polymeric micelles self-assembled from the copolymer, the hydrodynamic diameter, distribution and zeta potential of polymeric micelles in PBS solutions with different pH values (7.4, 6.0 and 5.0) were measured by DLS, as shown in figure 6. As shown in figure 6*a*, when the pH value of the copolymer solution was higher than 7.4, the particle size slowly increased with the increasing pH value. This was ascribed to there being little aggregation of the polymeric micelles under alkaline conditions. When the pH value decreased to acidic conditions, the particle sizes increased notably, from 215 nm (pH 7.4) to 336 nm (pH 6.0) and 428 nm (pH 5.0), respectively. The reason was that when the pH values were lower than the p*K*_b_ of the copolymer, the tertiary amine groups of PAE segment would be ionized leading to the transformation of the pH-sensitive PAE from hydrophobic to hydrophilic segments, resulting in swelling of the polymeric micelles. Similar changes are shown in figure 6*b*, which demonstrates that the curves shifted to the right and the polydispersity index (PDI) of the polymeric micelles was increased as pH decreased. As shown in figure 6*c*, the zeta potentials of the samples were negative under alkaline conditions due to the increase of hydroxyl groups in the solution. At the pH of 7.4, the zeta potential of the self-assembled polymeric micelles exhibited very low positive charge (+2 mV). When the pH values decreased to the acidic condition, the zeta potential was sharply enhanced (+24 or +29 mV at pH of 6.0 or 5.0, respectively), attributed to the full protonation of the tertiary amine residues of the copolymer. This result suggests that the polymeric micelles could undergo the charge reversal process when they accumulate at acidic sites, such as tumour and infection sites, leading to high cellular uptake because of the coulombic interaction between polymeric micelles and cells. Figure 6*d* shows a TEM image of the polymeric micelles at pH 7.4, which indicated that the polymeric micelles took a spherical morphology. The particle size was about 200 nm, which was smaller than that determined by DLS. This discrepancy could be due to shrinking of the particles during the drying process prior to TEM analysis and an intensity average presented by the DLS data.

The hydrophobic anti-cancer drug DOX was selected as model drug and encapsulated into the polymeric micelles by dialysis method. The drug loading efficacy and other characteristics are listed in table 1. As seen from the results, when the weight ratio of DOX to copolymer was 1 : 4, the LC and EE were 15.9% and 60.4%, respectively. As the ratio increased to 2 : 4, the LC was enhanced to 20.2% while the EE was decreased to 50.5%. Therefore, the feed ratio (1 : 4) of copolymer to drug was used in the following study. The hydrodynamic diameter of the DOX-PMs was slightly increased due to the encapsulated DOX molecules in the micellar core. The zeta potential of the DOX-PMs was also enhanced in comparison to that of the blank polymeric micelles because the DOX molecules showed positive charge.

**Table 1.** Characterization of DOX-PMs at pH 7.4.

copolymer (mg)	DOX (mg)	LC^a^ (%)	EE^a^ (%)	size^b^ (nm)	PDI^b^	zeta-potential^b^ (mV)
40	0	—	—	215.5	0.233	0.8
40	5	5.6	39.2	231.4	0.242	2.7
40	10	15.9	60.4	238.0	0.237	3.1
40	20	20.2	50.5	242.3	0.252	3.4

^a^Detected by the UV–vis.

^b^Detected by the DLS.

### 3.5. *In vitro* release of DOX from polymeric micelles

**Figure 7.**
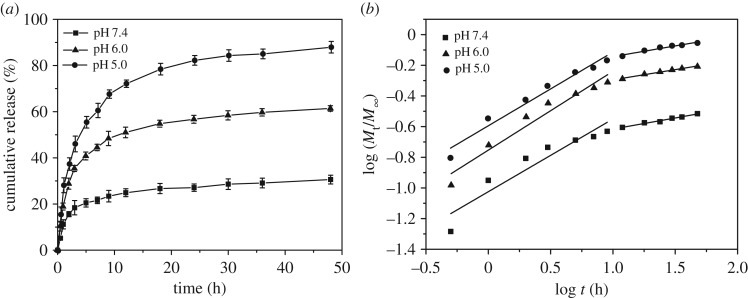
*In vitro* DOX release behaviour (*a*) and release mechanism (*b*) from DOX-PMs incubated in PBS solutions with pH of 7.4, 6.0 or 5.0.

To confirm the pH-triggered drug release behaviour of the DOX-PMs, the *in vitro* DOX release performance was evaluated in PBS solutions at pH 7.4 (normal physiological condition) or pH 6.0 and 5.0 (tumoral conditions), as shown in figure 7*a*. It was observed that the drug release from the DOX-PMs was dependent on the pH value and time. At pH 7.4, the drug release rate was slow and the cumulative releases were only 15.6%, 27.1% and 30.6% for 2, 24 and 48 h, respectively. The reason could be that the polymeric micelles maintained a tight and compact structure and entrapped the drug molecules in the core, preventing the release of drug from the pH-sensitive polymeric micelles. When the pH value decreased to a slightly acidic condition (pH 6.0), the cumulative releases were 28.7%, 56.6% and 61.3% for 2, 24 and 48 h, respectively. As mentioned above, the tertiary amine groups were ionized when the pH value was lower than the p*K*_b_ of the polymer, leading to the swelling of the polymeric micelles and acceleration of drug release rate. The swelling of the DOX-PMs indicates a larger porosity, suggesting that more drug molecules would be released from the micellar core. Additionally, the enhanced positive zeta potential also could strengthen the repulsive force between hydrophilic segments, which would also result in a more porous topological structure. When the pH value was 5.0, the drug release rate was significantly accelerated, and the cumulative releases were 37.5%, 82.3% and 88.9% for 2, 24 and 48 h, respectively. This was attributed to the loose and porous topological structure of the polymeric micelles resulting from the full ionization of the tertiary amine residues and the transformation of PAE segments from hydrophobic to hydrophilic.

To further investigate the drug release mechanism from the polymeric micelles, drug release behaviours were analysed by the comprehensive semi-empirical equations (3.1) and (3.2) established by Siepmann & Peppas [45]:

3.1 $$\frac{M_{t}}{M_{\infty }}=kt^{n}$$

and

3.2 $$\log\left(\frac{M_{t}}{M_{\infty }}\right)=n\log t+\log k,$$

where *M_t_*/*M*_∞_ was the percentage amount of cumulative drug release at time *t*. The constants *n* and *k* were drug release exponents: *n* indicated the release mechanism, and *k* was decided by the structural and geometric characteristics of the spherical nanoparticles. When *n* = 0.43 or 0.85, the drug release mechanism was Fichian diffusion or a swelling-controlled mechanism, respectively, and *n* < 0.43 or 0.43 < *n* < 0.85 corresponded to the combination of diffusion and erosion control mechanisms or anomalous transport mechanism, respectively.

In this study, the drug release process was divided into two stages, one from 0 to 12 h and the other from 12 h to 48 h. Figure 7*b* represents the theoretical fitted to experimental release data for DOX-PMs. The fitting parameters (release exponent *n* and rate constant *k*) at different time stages were tabulated in table 2. Figure 7*b* indicates the good linearity of fitting results. In the first stage, the release exponent *n* was 0.44 at pH 7.4, demonstrating that the drug release mechanism was Fichian diffusion. However, when the pH was acidic, the *n* values were 0.51 and 0.48, indicating that the drug release mechanism accorded with anomalous transport mechanism. In the second stage, the *n* values at different pH conditions were less than 0.43, suggesting that the DOX release behaviours corresponded to the combination of diffusion and erosion control mechanisms. Moreover, in the first stage, the *k* values were 0.09, 0.18 and 0.25 for pH 7.4, 6.0 and 5.0, respectively, displaying that the release rate was increased as pH decreased, which was consistent with the results of the *in vitro* experiment.

**Table 2.** Release exponent (*n*) and rate constant (*k*) of DOX-PMs at different pH values.

pH	*n* (0–12 h)	*k* (0–12 h)	*n* (12–48 h)	*k* (12–48 h)
7.4	0.44	0.09	0.16	0.17
6.0	0.51	0.18	0.13	0.37
5.0	0.48	0.25	0.14	0.52

In summary, drug release behaviour from the polymeric micelles was significantly influenced by the environmental pH conditions. The drug release rate and cumulative amount at acidic conditions were much higher in comparison with those at pH 7.4. Thus, the prepared DOX-PMs were able to effect a pH-triggered drug release.

### 3.6. Cytotoxicity test

**Figure 8.**
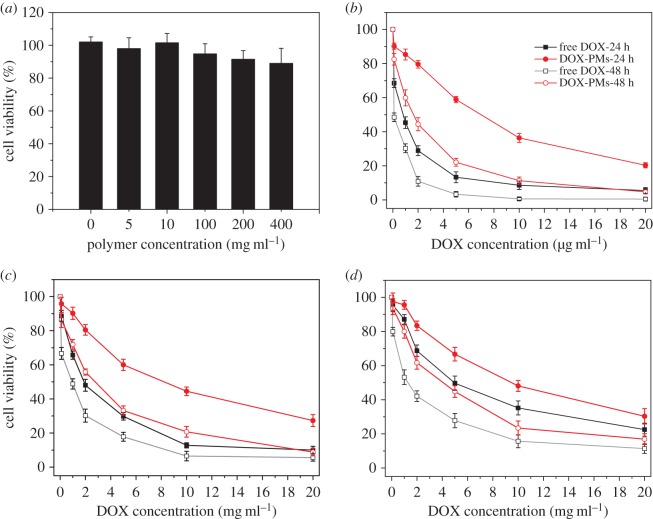
The cell viabilities of B16F10 cells incubated with various concentrations of blank polymeric micelles for 48 h (*a*), and the cell viabilities of B16F10 (*b*), HepG2 (*c*) and HeLa (*d*) cells incubated with free DOX or DOX-PMs for 24 or 48 h in concentration gradients.

Cytotoxic effects of the blank polymeric micelles, free DOX or DOX-PMs against B16F10, HepG2 and HeLa cells were evaluated by MTT assays, as shown in figure 8. As seen in figure 8*a*, the synthesized copolymer showed negligible cytotoxicity for B16F10 cells after incubation of 48 h. The cytotoxic effect of copolymer slightly increased with increasing polymer concentration, but the percentage of viable cells was higher than 90% even at the highest concentration of 400 mg l^−1^. This result demonstrated that the prepared copolymer revealed no significant cytotoxicity for B16F10 cells. Figure 8*b–d* presented the cytotoxicity of free DOX or DOX-PMs against B16F10, HepG2 and HeLa cell lines for 24 and 48 h, respectively. The IC50 values were 0.8 and 0.09 µg ml^−1^ (B16F10), 1.7 and 0.9 µg ml^−1^ (HepG2), 4.6 and 1.5 µg ml^−1^ (HeLa) for free DOX at 24 and 48 h, respectively. By contrast, the IC50 values were 6.0 and 1.5 µg ml^−1^ (B16F10), 9.2 and 2.6 µg ml^−1^ (HepG2), 10.5 and 3.6 µg ml^−1^ (HeLa) for DOX-PMs at 24 and 48 h, respectively. The results indicated that the DOX-PMs had lower cytotoxicity than that of free DOX for the same incubation time. The cytotoxicity of DOX-PMs against the tested tumour cell lines increased greatly with increasing drug concentration. This phenomenon could be because the release of DOX from the polymeric micelles was time dependent, resulting in lower drug concentration than that of free DOX in treated cells at the same time. Nevertheless, it was observed that DOX-PMs showed similar cytotoxic effect at the highest drug concentration (20 µg ml^−1^) with incubation time of 48 h compared with the free DOX, which indicated that the DOX could be released from the micelles, and the polymeric micelle envelope might not inhibit the cytotoxicity of encapsulated drug molecules.

## 4. Conclusion

The pH-responsive amphiphilic copolymer DSPE-*b*-PEG-*b*-PAE-*b*-PEG-*b*-DSPE was successfully synthesized *via* Michael-type polymerization, and its self-assembled polymeric micelles were prepared and used as carriers for the hydrophobic anti-cancer drug DOX. The copolymer had a low CMC value which showed the stability of the micelle and potential application in DDS. The polymeric micelles showed high drug loading capacity and entrapment efficacy. Furthermore, the prepared polymeric micelles exhibited pH-sensitivity. The hydrodynamic diameter, distribution and zeta potential were changed dependent on the pH value. The *in vitro* drug release experiment and theoretical simulation displayed that the drug release from the DOX-PMs was pH-triggered. The cytotoxicity of DOX-PMs against B16F10, HepG2 or HeLa cells was similar to that of free drug after 48 h incubation. The findings demonstrated that the synthesized copolymer and prepared pH-responsive polymeric micelles could be a potential anti-cancer drug delivery system for cancer chemotherapy.

## Data accessibility

There is no supporting material or special data because all equations, parameters used and algorithm are presented in the main text of the work and could be reproduced by anyone. Thus, the paper contains complete self-sufficient information and does not need any additional data files.
